# Human *Salmonella* Infection Yielding CTX-M β-Lactamase, United States

**DOI:** 10.3201/eid1412.080494

**Published:** 2008-12

**Authors:** Maria Sjölund, Jennifer Yam, Jillian Schwenk, Kevin Joyce, Felicita Medalla, Ezra Barzilay, Jean M. Whichard

**Affiliations:** Centers for Disease Control and Prevention, Atlanta, Georgia, USA (M. Sjölund, J. Yam, J. Schwenk, K. Joyce, F. Medalla, E. Barzilay, J.M. Whichard); Atlanta Research and Education Foundation, Atlanta (M. Sjölund, J. Yam, K. Joyce)

**Keywords:** Salmonella, antimicrobial drug resistance, CTX-M-5 beta-lactamase, letter

**To the Editor:** In the United States most third-generation cephalosporin resistance among salmonellae is due to AmpC plasmid–mediated β-lactamases. Extended-spectrum β-lactamases (ESBLs) have rarely been reported (*1*). The CTX-M β-lactamases constitute a group of ESBL enzymes that are increasing in prevalence worldwide. Currently, the CTX-M enzymes are classified into 5 different subgroups on the basis of DNA sequence similarities (*2*). We report on a domestically acquired CTX-M­–producing *Salmonella* isolate in the United States.

In 2003, public health laboratories in all US state health departments submitted every 20th non-Typhi *Salmonella* (NTS) isolate from humans to the Centers for Disease Control and Prevention (CDC) for susceptibility testing by the National Antimicrobial Resistance Monitoring System (NARMS). MICs were determined by broth microdilution and interpreted according to Clinical and Laboratory Standards Institute standards (www.clsi.org), when available. Resistance to cefquinome was defined as >32 mg/L.

Among the 1,864 human NTS isolates submitted to NARMS in 2003, 105 (5.6%) displayed elevated MICs (>2 mg/L) to ceftriaxone or ceftiofur, third-generation cephalosporins used in human and veterinary medicine, respectively. Genomic DNA was prepared from the 105 isolates, and a PCR with degenerate primers capable of detecting all CTX-M enzymes identified a single positive *S. enterica* ser. Typhimurium (*3*). The isolate came from a stool sample collected in September 2003 from a white, non-Hispanic, US-born, 3-month-old boy who lived in the state of Georgia. The patient had diarrhea and fever for ≈1 week. Because neither the patient nor his family had traveled internationally in the 3 months before specimen collection, the infection appears to have been domestically acquired. The patient did not receive any antimicrobial agents before illness but was treated for 14 days with cefpodoxime. The infant recovered from the illness without complications.

The isolate displayed resistance to β-lactams, aminoglycosides, phenicols, tetracyclines, and folate pathway inhibitors (Table). Two β-lactamases (isoelectric pH [pI] 7.5 and 8.8) were resolved by isoelectric focusing.

**Table:** MIC values of antimicrobial drugs for the *Salmonella* ser. Typhimurium isolate and its *Escherichia coli* DH10B transformant

Antimicrobial agent	MIC, mg/L		
	*S.* ser. Typhimurium	*E. coli* DH10B transformant	*E. coli* DH10B
Amikacin	1	1	1
Amoxicillin–clavulanic acid	32	16	4
Ampicillin	>32	>32	4
Aztreonam	32	32	0.12
Cefepime	32	32	<0.06
Cefotaxime	>64	>64	<0.06
Cefotaxime–clavulanic acid	0.25	0.12	<0.06
Cefoxitin	2	8	8
Cefquinome	>32	>32	<0.06
Ceftazidime	8	8	0.5
Ceftazidime–clavulanic acid	0.5	0.25	0.12
Ceftiofur	>8	>8	0.5
Ceftriaxone	>64	>64	<0.25
Chloramphenicol	>32	<2	<2
Ciprofloxacin	<0.016	<0.016	<0.016
Gentamicin	4	<0.25	<0.25
Imipenem	0.5	0.25	0.25
Kanamycin	>64	<8	<8
Nalidixic acid	4	1	1
Piperacillin-tazobactam	>64	2	2
Streptomycin	<32	>64	>64
Sulfisoxazole	>256	<16	<16
Tetracycline	>32	<4	<4
Trimethoprim-sulfamethoxazole	>4	<0.12	<0.12

Group-specific PCR primers were used to characterize the presumed *bla*_CTX-M_ gene (*4*). Primers TOHO1–2F and TOHO1–1R yielded a 351-bp product, confirming a group II *bla*_CTX-M_ gene. To perform sequencing of the entire gene, a ClustalW alignment with representatives from group II was performed to identify primers (DNASTAR, Madison, WI, USA). The sequence of the gene was identical to the sequence of the *bla*_CTX-M-5_ gene detected in other isolates of *S. enterica* ser. Typhimurium (GenBank accession nos. U95364 and AF286192) as well as to the *kluA-2* gene of *Kluyvera ascorbata* (GenBank accession no. AJ251722).

The genetic environment of the *bla*_CTX-M-5_ gene was investigated by PCR specific for upstream insertion elements (IS*Ecp1*, IS*26*, and ORF513) and the downstream sequence *sul1* (*5*). Amplification with primer IS*Ecp1* and an internal *bla*_CTX-M-5_ primer yielded a PCR product of ≈350 bp. Sequencing confirmed presence of the 3′ end inverted repeat region of the IS*Ecp1*.

Presence of other β-lactamase–encoding genes (*bla*_TEM_, *bla*_SHV_, and *bla*_OXA_) was investigated by PCR (*6*–*8*). Amplification with primers OXA-1F and OXA-1R yielded a 595-bp product with a sequence consistent with that of *bla*_OXA-1_ (*8*).

To determine whether the CTX-M enzyme was plasmid-borne, plasmids were extracted and transformed into electrocompetent *Escherichia coli* DH10B. The transformant exhibited resistance to cefotaxime but not to ceftazidime (Table). In addition, the transformant exhibited resistance to cefquinome and cefepime. The presence of a *bla*_CTX-M_ gene was confirmed by PCR (*3*,*4*). The *bla*_OXA_ gene could not be amplified from the *E. coli* transformant (*8*).

A CTX-M–producing *Salmonella* isolate has been reported only once previously in the United States (*9*). This was in 1994, when an isolate of *Salmonella* ser. Typhimurium var. Copenhagen with a CTX-M-5 was recovered from a 4-month-old girl adopted from Russia; that infection was not domestically acquired (*9*). We compared the 1994 isolate and the isolate in this study by pulsed-field gel electrophoresis; the isolates showed distinct patterns.

The IS*Ecp1* insertion sequence has been described as a flanking region of several *bla*_CTX-M_ genes and has been implicated in the expression and mobilization of the genes (*5*). A recent study by Lartigue et al. showed that a CTX-M-2 progenitor in *K. ascorbata* could be mobilized and transferred to a conjugative *E. coli* plasmid by the IS*Ecp1B* element; enhanced mobilization was observed in the presence of ceftazidime, cefotaxime, and piperacillin (*10*).

This *Salmonella* isolate’s resistance to cefepime and cefquinome, fourth-generation cephalosporins, is troubling. Cefquinome is not approved for use in the United States but has been used in Europe for treating food animals since 1994. ESBLs, including CTX-M enzymes, are more common in Europe than in the United States (*1*). Further studies are warranted to clarify the extent to which the use of cefquinome has contributed to high CTX-M prevalence in Europe.

In conclusion, we report a domestically acquired CTX-M–producing *Salmonella* isolate in the United States. Because third-generation cephalosporins are important for treating invasive *Salmonella* infections, continued monitoring of ESBL-producing bacteria is important.
